# Assessing protein energy wasting in a Malaysian haemodialysis population using self-reported appetite rating: a cross-sectional study

**DOI:** 10.1186/s12882-015-0073-x

**Published:** 2015-07-07

**Authors:** Sharmela Sahathevan, Chee Hee Se, See Hoe Ng, Karuthan Chinna, Gilcharan Singh Harvinder, Winnie Siew Swee Chee, Bak Leong Goh, Halim A. Gafor, Sunita Bavanandan, Ghazali Ahmad, Tilakavati Karupaiah

**Affiliations:** Dietetics Program, School of Healthcare Sciences, Faculty of Health Sciences, National University of Malaysia, Jalan Raja Muda Abdul Aziz, 50300 Kuala Lumpur, Malaysia; Epidemiology and Biostatistics Unit, Department of Social and Preventive Medicine, Faculty of Medicine, University of Malaya, 50603 Kuala Lumpur, Malaysia; Division of Nutrition and Dietetics, School of Health Sciences, International Medical University, No.126, Jalan Jalil Perkasa 19, Bukit Jalil, 57000 Kuala Lumpur, Malaysia; Department of Nephrology, Serdang Hospital, Jalan Puchong, 43000 Kajang, Selangor Malaysia; Nephrology Unit, Department of Medicine, Universiti Kebangsaan Malaysia Medical Centre, Jalan Yaacob Latif, Bandar Tun Razak, Cheras, Kuala Lumpur, 56000 Malaysia; Department of Nephrology, Kuala Lumpur Hospital, Jalan Pahang, 50586 Kuala Lumpur, Malaysia

**Keywords:** Appetite, Haemodialysis, Protein energy wasting, Nutritional status, Anorexia

## Abstract

**Background:**

Poor appetite could be indicative of protein energy wasting (PEW) and experts recommend assessing appetite in dialysis patients. Our study aims to determine the relationship between PEW and appetite in haemodialysis (HD) patients.

**Methods:**

HD patients (n=205) self-rated their appetite on a scale of 1 to 5 as *very good* (1), *good* (2), fair (3), *poor* (4) or *very poor* (5). Nutritional markers were compared against appetite ratings. Using logistic regression analysis associations between dichotomized appetite with PEW diagnosis were determined as per the International Society of Renal Nutrition and Metabolism (ISRNM) criteria and alternate objective measures. Data was adjusted for socioeconomic and demographic characteristics.

**Results:**

Poorer appetite ratings were significantly associated with lower income (*P* = 0.021), lower measurements (*P* < 0.05) for mid-arm muscle circumference, mid-arm muscle area and lean tissue mass (LTM), serum urea (*P* = 0.007) and creatinine (*P* = 0.005). The highest hsCRP (*P* = 0.016) levels occurred in patients reporting the poorest appetite. Serum albumin did not differ significantly across appetite ratings. Poor oral intake represented by underreporting (EI/BMR < 1.2) was evident for all appetite ratings. PEW was prevalent irrespective of appetite ratings (*very good*: 17.6 %, *good*: 40.2 %, *fair*: 42.3 % and *poor*: 83.3 %). After dichotomizing appetite ratings into normal and diminished categories, there was a marginal positive association between diminished appetite and overall PEW diagnosis (OR_adj_: 1.71; 95 % CI: 0.94–3.10, *P* = 0.079). Amongst individual ISRNM criteria, only BMI <23 kg/m2 was positively associated with diminished appetite (OR_adj_: 2.17; 95 % CI: 1.18–3.99). However, patients reporting diminished appetite were more likely to have lower LTM (OR_adj_: 2.86; 95 % CI: 1.31–6.24) and fat mass (OR_adj_: 1.91; 95 % CI: 1.03–3.53), lower levels of serum urea (OR_adj_: 2.74; 95 % CI: 1.49–5.06) and creatinine (OR_adj_: 1.99; 95 % CI: 1.01–3.92), higher Dialysis Malnutrition Score (OR_adj_: 2.75; 95 % CI: 1.50–5.03), Malnutrition Inflammation Score (OR_adj_: 2.15; 95 % CI: 1.17–3.94), and poorer physical (OR_adj_: 3.49; 95 % CI: 1.89–6.47) and mental (OR_adj_: 5.75; 95 % CI: 3.02–10.95) scores.

**Conclusions:**

A graded but non-significant increase in the proportion of PEW patients occurred as appetite became poorer. However, after dichotomization, a positive but marginally significant association was observed between diminished appetite and PEW diagnosis.

**Electronic supplementary material:**

The online version of this article (doi:10.1186/s12882-015-0073-x) contains supplementary material, which is available to authorized users.

## Background

Survival for most end stage renal disease patients in Malaysia is by means of maintenance haemodialysis (HD). As of December 2013, there were 28,822 patients undergoing maintenance HD in Malaysia which represents a 3-fold exponential increase over the past decade [1]. Annual figures from the Malaysian Dialysis and Transplant Registry cite malnutrition as a major problem as indicated by ~60 % of the HD population having serum albumin concentrations ≤40 g/L and body mass index (BMI) ≤25 kg/m^2^ [2]. These figures have persisted since 2002 [2]. Unfortunately, although poor oral intake in these patients is the likely major factor contributing to malnutrition, the lack of dietician access to probe this issue prevails in most dialysis centres in Malaysia [3].

Diminished appetite is often implicated in the chronology of protein-energy malnutrition which is prevalent in 30 to 75 % of the HD population [4]. However, the emerging complex face of malnutrition today is protein energy wasting (PEW) syndrome and poor oral intake is hypothesized as one of the contributory factors. PEW patients commonly experience multiple nutritional and catabolic alterations that encompass persistent inflammation, acidosis and a state of hyper-metabolism leading to catabolism of muscle and fat [5]. These are facets that are also common to anorexia and cachexia aetiology in chronic disease [6]. Thus, assessment of under-nutrition remains problematical in PEW patients because of underreporting of diet records and the lack of trained dietary skills to quantify energy and protein intake [5]. Expert opinion suggests that appetite assessment may serve as a diagnostic tool for PEW in dialysis patients [7].

No studies to date have explored the link between appetite and PEW. Appetite assessment in HD populations were carried out using the single question pertaining to appetite from the 44-item Appetite and Diet Assessment Tool (ADAT), originally developed by the Haemodialysis Study Group [8]. Studies have used this single question to correlate appetite with nutritional status of HD populations in different regions such as United States [8, 9], Italy [6] and Australia [10]. Responses to this question- *During the past one week, how would you rate your appetite?* are ranked according to 5 ratings, inclusive of *very good, good, fair, poor* and *very poor*. In a study comparing dietary intakes on dialysis and non-dialysis days, researchers concluded that appetite assessment using this 5-scaled question enabled detection of patients who had poor appetite requiring early intervention [9]. Bossola et al. [6] using the same appetite question found that appetite may fluctuate over time that could be associated with older age, more co-morbidities and frequent hospitalizations.

Since appetite assessment is widely accepted as an early warning of impending morbidity and nutritional concerns [11, 12], we were interested in examining the relationship between the appetite question with the nutritional status and diagnosis of PEW in a Malaysian HD population. A recently expressed concern was the need to understand socioeconomic factors mediating cultural and environmental determinants of health outcomes in the chronic kidney disease population [13]. In this context, it would be interesting to see if socio-cultural and multi-ethnic differences in these patients would affect the usability of this self-reported appetite rating. We hypothesize that self-reported appetite if correlated to markers of nutritional status, will also correlate with PEW diagnosis in the Malaysian HD population.

## Methods

### Study design and patient recruitment

**Fig. 1:**
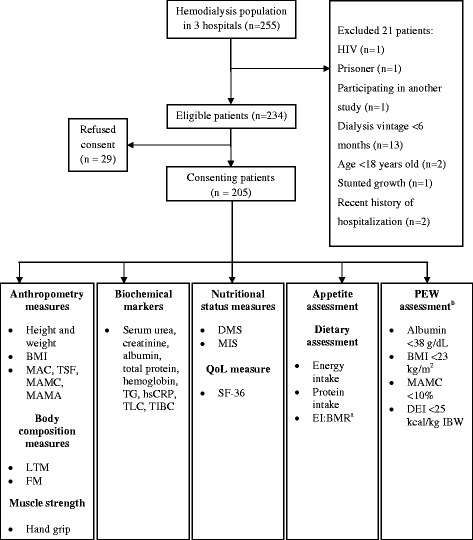
Study flow of participants. Abbreviations: BMI = Body Mass Index; DEI = dietary energy intake; DMS = Dialysis Malnutrition Score; EI:BMR = energy intake to basal metabolic rate ratio; FM = fat mass; HIV = human immunodeficiency virus; hsCRP = high sensitivity C-reactive protein; IBW = ideal body weight; LTM = lean tissue mass; MAC = mid-arm circumference; MAMA = mid-arm muscle area; MAMC = mid-arm muscle circumference; MIS = Malnutrition Inflammation Score; PEW = protein energy wasting; QoL = Quality of life; SF-36 = short-form (36-item) questionnaire; TG = triglyceride; TIBC = total iron binding capacity; TLC = total lymphocyte count; TSF = triceps skinfold. ^*a*^ EI:BMR cut-offs based on Black [27]. ^*b*^ PEW assessment based on ISRNM diagnostic criteria [7]

This cross-sectional study was part of a baseline screening protocol for an oral protein supplementation program which aimed at recruiting malnourished HD patients [14]. Patient recruitment was conducted between February 2011 to May 2012 from HD units at two government tertiary referral hospitals and one teaching hospital in the Klang Valley, with a combined patient pool of 255. Initial recruitment criteria were inclusive of patients dialyzing for ≥6 months, aged ≥18 years, clinically stable, able to consume food orally, not dysphagic, able to self-report appetite in either language- Malay or English and provide written consent. Patients with cognitive impairment or terminal illnesses such as HIV/AIDS or malignancy were excluded during the recruitment period. Additional exclusion criteria included patients with repeated history of hospitalization or inter-current illnesses in the six months prior to the recruitment. A total of 205 patients consented to participate, giving a response rate of 80.4 %. However, two subjects refused to participate for body composition analysis. The stock flow of patients included in the final analysis is presented in Fig. 1. This study was approved by the Medical Research and Ethics Committee, Ministry of Health, Malaysia (NMRR-11-355-9148) and Medical Research Ethics Committee of National University of Malaysia (FF-274-2012).

### Nutritional status assessments

All evaluations were synchronized with routine blood collection dates of patients. Demographic data and measurement of dialysis dose (Kt/V) were obtained from patients’ medical records.

#### Appetite assessment

Patients’ appetite was assessed using the first question from the original 44-item ADAT used in the Haemodialysis Study Group [8]. It was a single, self-administered question with multiple-choice responses: *During the past week (7 days), how would you rate your appetite?* Patients were required to indicate their responses using a scale of 1 to 5: *1)* very good, *2)* good, *3)* fair, *4)* poor or *5)* very poor. The question was administered to patients in either language- Malay or English. The English version was translated into the Malay language by three researchers (SS, CHS, SHN) who are native speakers of the Malay language. The Malay question read as, *Dalam tujuh hari yang lepas, bagaimanakah selera makan anda?* 1) *Amat baik* (very good), 2) *baik* (good), 3) *kadang-kala baik, kadang-kala kurang baik* (fair), 4) *kurang baik* (poor) and 5) *amat kurang baik* (very poor).

#### Anthropometric and body composition evaluations

Measurements of weight (pre- and post-dialysis) and height were taken using a SECA digital scale (Model 220, SECA, Germany) to derive BMI [in kg/m^2^; weight (kg)/ height (m^2^)]. Triceps skinfold thickness (TSF) measurement was taken on the non-fistula arm using a Harpenden skinfold calliper (HSK-BI, British Indicators, West Sussex, UK). Mid-upper arm circumference (MAC) was measured using a non-stretch Lufkin® metal measuring tape (Apex Tool Group, LLC, NC, USA). Procedures for TSF and MAC were conducted as per the protocol outlined by the International Society for the Advancement of Kinanthropometry [15]. The mid-arm muscle circumference (MAMC) and mid-arm muscle area (MAMA) were calculated using the following equations [16]:

$$ MAMC\ (cm) = MAC\ (cm)\ \mathit{\hbox{--}}\ \left[TSF\ (cm)\ x\ \pi \right] $$

$$ MAMA\ \left(c{m}^2\right) = MAMC\ {(cm)}^2/ 4\pi\ \mathit{\hbox{--}}\  10.0\ \left(for\  men\right)\  or\  6.5\ \left(for\  women\right) $$

Hand grip strength test, as a surrogate measure of muscle strength, was assessed before patients initiated their dialysis session. The hand grip strength test was carried out using the Jamar dynamometer (BK-7498; Fred Sammons, Inc., Burr Ridge, IL) on the non-fistula hand. Three readings were taken and the median value was used. All anthropometric measurements were performed by a trained dietician to eliminate inter-observer variation.

Body composition and hydration status were assessed using a portable whole-body bio-impedance spectroscopy device (Body Composition Monitor, Fresenius Medical Care, Bad Homburg, Germany). The use of this tool in assessing the hydration status in Malaysian HD population has been reported elsewhere [17]. Body composition measurements were carried out before the dialysis session, with the patient resting in the supine position for about 15 min prior to the measurement. The electrodes were placed on the wrist of the non-fistula arm and on the ipsilateral ankle and subsequently connected to the device [18]. The hydration status, lean tissue mass (LTM) and fat mass (FM) generated by the instrument were based on a physiologic tissue model as described by Chamney et al. [19].

#### Laboratory investigations

Serum urea (by urease-glutamate dehydrogenase method), creatinine (by Jaffe method), total iron-binding capacity, haemoglobin (by colorimetric method), albumin (by bromocresol green method), total protein, triglycerides (by enzymatic methods) and total lymphocyte count (by nephelometry) were analysed using an automated clinical chemistry analyser (Roche/Hitachi 912 System, Roche Diagnostics, Tokyo, Japan). The analyses were carried out as per the in-house standard operating procedures endorsed by the Ministry of Health, Malaysia. As serum high-sensitivity C-reactive protein (hsCRP) was not routinely performed, it was measured by nephelometric turbidimetric immunoassay at an independent laboratory [20, 21]. The lower detection limit of hsCRP was 0.03 mg/L and the mean intra-assay coefficient of variation was less than 3 % for the automated analysis.

#### Dietary assessment

Patients were required to provide 24-h dietary records for three days inclusive of a dialysis day, a non-dialysis day and one optional weekend day, as suggested by Fouque et al. [22]. To minimize error in this data collection, patients first received familiarization training from trained research dieticians thus enabling reported food portions to be scaled according to standard household measurements. Food intake records were analysed for nutrient intake using the Nutritionist Pro software (Nutritionist Pro™ 2.2.16, First DataBank Inc., 2004) which included ethnic-specific Malaysian foods [23, 24]. Dietary energy and protein intakes were then interpreted in terms of patients’ ideal body weight. Energy over- and under-reporting were identified using cut-off points based on the reported energy intake to basal metabolic rate (EI:BMR) ratio [25]. Patients’ basal metabolic rate was estimated using the Harris-Benedict equation [26]. Patients scoring EI:BMR ratios of <1.2, 1.2–2.4 and >2.4 were classified as under-, acceptable and over-reporters of energy intake respectively [27].

#### Nutritional screening tools

Two nutritional screening tools, namely Dialysis Malnutrition Score (DMS) and the Malnutrition Inflammation Score (MIS), which focus on measuring the severity of malnutrition-inflammation complex syndrome, were administered in these patients during dialysis sessions. The DMS consisting of the 7 original Subjective Global Assessment components (*weight change, dietary intake, gastrointestinal symptoms, functional capacity, disease and physical examination for signs of muscle and fat wasting*) provides a 5-point scale ranging from 1 (*normal*) to 5 (*very severe malnutrition*) [28]. Cumulative scores from total assessment range from 7 to 35 with rating varying from normal to moderate to severe malnutrition. MIS evaluation is based on the 7 components of Subjective Global Assessment with the addition of BMI, serum albumin and total iron binding capacity. Each component is scored using a 4-point scale with 0 (*normal*) to 3 (*very severe*) [29]. The cumulative score ranges from 0 (*normal*) to 30 (*severely malnourished*).

#### Quality of Life (QoL)

QoL was assessed using an interviewer-administered 36-Item Short Form Health Survey (SF-36) questionnaire exploring 8 domains of general health construct, namely physical functioning, role limitations caused by physical problems, bodily pain, general health perceptions, vitality, social function, role limitation caused by emotional problems and general mental health [30]. This tool has been applied and well validated for different diseased populations with different cultural backgrounds including Asian populations [31–34]. Patients were required to report their functional and mental health status in the preceding 4 weeks using a Likert-scale. A scoring algorithm was used to recode the selected scale into scores ranging from 0 to 100. The obtained scores were assigned to the respective domains and the scores were averaged. Domains were summarized into 2 scales [a] the physical component scale as a measure of physical health and [b] the mental component scale as a measure of emotional function. The sum total of both domains provided the scoring for the total SF-36 [35]. Higher scores indicated better health in HD patients.

#### PEW assessment

PEW was identified in patients based on diagnostic criteria provided by the International Society of Renal Nutrition and Metabolism (ISRNM) expert panel [7]. The PEW diagnosis consists of 4 main categories: biochemical assessment, low body weight, reduced total body fat or weight loss, decreased muscle mass and low energy or protein intake. Any 3 of the 4 established criteria had to be met in order to be diagnosed as PEW. In our study, patients were assessed for serum albumin <38 g/dL, BMI <23 kg/m^2^, reduction >10 % in MAMC in relation to 50th percentile of reference population and dietary energy intake <25 kcal/kg ideal body weight. In addition, we also assessed patients for potential markers of PEW as proposed by ISRNM in terms of body mass and composition measures: LTM and FM, laboratory markers: serum urea, creatinine, triglyceride, total lymphocyte count, hsCRP and nutritional status screening tools (DMS and MIS). We proposed to include hand grip strength and QoL (SF-36) as additional markers for PEW, as both are also recognized as measures of nutritional status [11, 35]. As dialysis-specific reference values for these markers were not available, we used the median value of each parameter as the cut-off limit.

### Statistical analysis

Variables are presented as frequency (percentages), mean ± SD or median with interquartile range. The normal distribution of continuous variables was assessed using Kolmogorov-Smirnov test. Differences between groups were analysed using one-way ANOVA for normally distributed continuous data whilst skewed data were analysed using Kruskal-Wallis test followed by Dunn’s *post-hoc* evaluation. Categorical variables were evaluated for association using Pearson *χ*^2^ test. Results were further tabulated into 2 × 2 contingency tables to determine the relationship between appetite and traditional PEW diagnostic criteria as well as potential markers of PEW, as proposed by Fouque et al. [7]. The odds ratio for diminished appetite was adjusted for confounding variables (age, gender, ethnicity, income level, co-morbidities and dialysis vintage) by logistic regression analysis. All analyses were computed using the IBM Statistical Package for Social Sciences version 19.0 (IBM SPSS Statistics Inc. Chicago IL. USA). Statistical significance was set at *P* < 0.05 for all evaluated parameters.

## Results

### Patient characteristics as per appetite ratings

A total of 205 HD patients had participated in this study. Patient characteristics are presented in Table 1. Our study patients had a slight majority of men (58 %) with an equal distribution of Malay (44.6 %) and Chinese ethnicities (44.1 %), while the remaining 11.4 % were Indians. The mean age of these patients was 52 ± 14 years and on average dialyzing for 7.9 ± 6.2 years. More than half the patients had hypertension (76.1 %), followed by diabetes (31.7 %) and cardiovascular disease (22.0 %). Majority of the patients (71.7 %) lacked an income and were financially dependent on their families. The mean Kt/V amongst these patients was 1.82 ± 0.44, which was higher than the recommended value of >1.2 [36]. Patients’ income was significantly associated with subjective appetite ratings (*P* = 0.021). Dialysis vintage years significantly differed across appetite ratings (*P* = 0.025).

**Table 1:** Characteristics of 205 haemodialysis patients

Demographic variables	Overall^a^	Self reported appetite ratings				P-value^b^
		Very good (n = 34)	Good (n = 87)	Fair (n = 78)	Poor (n = 6)	
**Gender**						
Male	119(58)	24 (70.6)	53 (60.9)	39 (50)	3 (50)	NS^c^
Female	86 (42)	10 (29.4)	34 (39.1)	39 (50)	3 (50)	
**Ethnicity** ^d^						
Malay	90 (44.6)	17 (50.0)	33 (38.4)	36 (47.4)	4 (66.7)	NS
Chinese	89 (44.1)	13 (38.2)	44 (51.2)	30 (39.5)	2 (33.3)	
Indian	23 (11.4)	4 (11.8)	9 (10.5)	10 (13.2)	0	
**Income**						
<RM1000	147 (71.7)	17 (50.0)	66 (75.9)	60 (76.9)	4 (66.7)	0.021
>RM1000	58 (28.3)	17 (50.0)	21 (24.1)	18 (23.1)	2 (33.3)	
**Co-morbidities**						
Hypertension	156 (76.1)	27 (79.4)	67 (77.0)	57 (73.1)	5 (83.3)	NS
Diabetes	65 (31.7)	12 (35.3)	27 (31.0)	23 (29.5)	3 (50.0)	NS
CVD	45 (22)	9 (26.5)	19 (21.8)	16 (20.5)	1 (16.7)	NS
Age (years)	51.8 ± 13.9	51.2 ± 11.8	51.4 ± 14.3	52.3 ± 14.8	55.8 ± 8.3	NS
Dialysis vintage (years)	7.9 ± 6.2	8.4 ± 6.4	6.6 ± 5.1	9.4 ± 6.9	5.7 ± 3.6	0.025
Kt/V^e^	1.82 ± 0.44	1.71 ± 0.46	1.83 ± 0.44	1.85 ± 0.43	1.90 ± 0.41	NS

^a^Data expressed as mean ± SD for continuous data; n (%) for categorical data

^b^Frequency data was analysed by Pearson χ^2^ test whilst continuous data was analysed by ANOVA

^c^NS = not significant

^d^Only for 202 patients as remaining 3 belonged to minor ethnic denominations

^e^Dialysis dose

### Nutritional markers as per appetite ratings

The appetite question had 5 scales. The *poor* and *very poor* ratings were grouped as *poor* due to the small patient number (Table 2). By this categorization, 34 (16.6 %) patients reported their appetite as *very good*, 87 (42.4 %) as *good*, 78 (38.1 %) as *fair* and 6 (2.9 %) as *poor*. Anthropometric measurements, serum biochemical markers, dietary intakes, nutritional status and QoL measures were compared across the appetite ratings for all 205 patients. Measures of muscle mass such as MAMC, MAMA and LTM were significantly lower in patients who reported *poor* appetite (*P* < 0.05) but not with hand grip strength (*P* > 0.05). A negative trend was noted for serum urea and creatinine with poorer appetite ratings from *very good* to *poor* (*P* < 0.05). For inflammatory status, patients reporting poor appetite had significantly greater hsCRP as compared to the other 3 groups. Patients had significantly lower total energy and protein intake when poor appetite was reported. Under-eating according to recommendations by KDOQI Guidelines was indicated when intakes were adjusted according to the ideal body weight [36]. Irrespective of all appetite ratings, the EI:BMR ratio was lower than the recommended value of >1.2 where 68.3 % (*n* = 140) of patients were under-reporters and 31.7 % (*n* = 65) were acceptable reporters. Increasing trends were noted for DMS (*P* < 0.001) and MIS (*P* < 0.001) with better appetite ratings, while there was a decreasing trend for SF-36 QoL scores (*P* < 0.001).

**Table 2:** Nutritional markers as per appetite ratings

Variable	Overall (Mean ± SD)^b^	Self-reported appetite ratings				P-value^c^
		Very Good (n = 34) Median (IQR)	Good (n = 87) Median (IQR)	Fair (n = 78) Median (IQR)	Poor^a^ (n = 6) Median (IQR)	
**Anthropometry and physical status**						
Weight (kg)	58.51 ± 14.0	68.50^d, e^ (54.40–72.00)	58.40^d, f^ (49.10–66.90)	54.40^e, f^ (45.98–60.50)	54.20 (45.33–62.28)	<0.001
BMI (kg/m^2^)	23.16 ± 4.71	25.80^d, e^ (23.26–27.30)	22.49^d^ (19.47–26.76)	21.76^e^ (19.11–24.85)	21.12 (19.30–24.46)	0.002
MAC (cm)	27.06 ± 3.77	28.55^d^ (24.98–30.23)	28.60^e^ (24.80–30.85)	25.85^d, e^ (23.38–28.35)	25.25 (22.24–27.40)	0.003
TSF (mm)	14.04 ± 5.95	14.00^d^ (11.58–19.58)	13.00 (9.70–18.70)	11.95^d^ (8.90–16.75)	12.50 (9.17–14.30)	NS
MAMC (cm)	22.83 ± 3.66	23.76^d^ (21.58–25.63)	23.09^e^ (20.79–26.13)	21.02^d, e^ (19.52–24.40)	19.53 (18.51–23.98)	0.017
MAMA (cm^2^)	33.74 ± 13.62	34.89^d^ (27.77–42.27)	32.50^e^ (25.18–44.32)	28.23^d, e^ (22.53–37.58)	23.01 (19.47–36.05)	0.032
LTM (kg)	31.14 ± 9.18	35.30^d, e^ (29.53–37.53)	30.70^f^ (23.95–39.85)	28.10^d, f^ (23.05–33.85)	24.90^e^ (22.93–30.83)	0.004
FM (kg)	19.89 ± 9.56	23.05 (14.75–28.58)	19.40 (11.15–26.05)	16.60 (13.08–24.23)	18.05 (11.35–25.73)	NS
HGS (kg)	16.91 ± 7.79	17.00 (12.00–22.50)	16.00 (12.00–24.00)	16.00 (10.00–20.00)	13.00 (8.00–16.00)	NS
**Serum chemistry**						
Urea (mmol/L)	19.55 ± 6.27	20.10^d^ (16.30–24.93)	19.70^e, f^ (17.30–23.50)	18.05^e, g^ (15.30–23.13)	15.10^d, f, g^ (9.60–16.75)	0.007
Creatinine (μmol/L)	864 ± 238	903^d, e^ (801–1121)	901^f, g^ (743–1078)	822^d, f^ (690–946)	743^e, g^ (505–833)	0.005
Albumin (g/L)	37.87 ± 4.72	38.00 (34.50–41.00)	38.00 (35.00–42.00)	37.00 (34.75–41.00)	35.00 (32.25–42.25)	NS
Total protein (g/L)	75 ± 6	76 (72–82)	75 (70–78)	74 (71–79)	76 (67–79)	NS
Hemoglobin (g/dL)	10.96 ± 1.71	10.70 (9.45–12.10)	11.40 (10.10–12.30)	10.8 (9.90–11.83)	10.45 (7.18–12.70)	NS
hsCRP (mg/L)	8.24 ± 17.92	3.34^d^ (1.92–5.64)	4.63^e^ (1.16–8.48)	3.16^f^ (1.33–7.89)	15.17 ^d, e, f^ (10.18–65.05)	0.016
**Dietary assessment**						
Energy (kcal/day)	1390 ± 384	1617^d ,e, f^ (1222–1766)	1408^d, g^ (1151–1638)	1311^e, h^ (1084–1552)	857^f, g, h^ (599–1340)	0.002
Protein (g/day)	56.17 ± 21.26	61.91^d, e, f^ (50.92–76.51)	54.28^d, g^ (43.27–67.65)	51.72^e, h^ (38.24–70.31)	29.5^f, g, h^ (19.92–36.80)	0.001
Energy (kcal/kg IBW)	23.12 ± 6.94	22.81^d^ (18.02–27.18)	21.98^e^ (18.57–27.22)	23.62^f^ (18.43–27.53)	14.34 ^d, e, f^ (11.69–19.82)	0.049
Protein (g/day IBW)	0.94 ± 0.39	0.89^d^ (0.65–1.25)	0.85^e^ (0.69–1.08)	0.87^f^ (0.66–1.19)	0.45^d, e, f^ (0.39–0.65)	0.010
EI:BMR	1.07 ± 0.30	1.03^d^ (0.84–1.30)	1.02^e^ (0.86–1.30)	1.07^f^ (0.87–1.24)	0.66^d, e, f^ (0.54–0.89)	0.039
**Nutritional status measures**						
DMS score	11.91 ± 2.66	10.50^d, f^ (10.00–13.00)	11.00^e, g^ (10.00–13.00)	12.00^d, e^ (10.00–14.00)	14.00^f, g^ (12.50–18.25)	<0.001
MIS score	6.55 ± 3.22	4.50^d, f^ (3.00–6.25)	6.00^e, g^ (4.00–8.00)	7.00^d, e^ (5.00–9.00)	9.50^f, g^ (7.00–11.50)	<0.001
**Quality of life measures**						
SF-36 PCS	64.36 ± 16.65	68.70^d^ (60.25–75.50)	75.00^e^ (60.00–80.00)	61.25^d, e^ (47.75–68.70)	69.35 (28.50–74.25)	<0.001
SF-36 MCS	70.42 ± 15.16	76.95^d, f^ (71.53–84.50)	78.00^e^ (67.00–83.40)	68.15^d, e^ (54.92–74.00)	69.27^f^ (60.90–73.45)	<0.001
SF-36 Total score	70.49 ± 15.67	76.16^d^ (71.20–82.22)	80.04^e^ (66.92–85.25)	64.66^d, e^ (53.44–75.85)	74.07 (49.78–79.88)	<0.001

Abbreviations: *BMI* Body Mass Index; *DMS* Dialysis Malnutrition Score; *EI:BMR* energy intake to basal metabolic rate ratio; *FM* fat mass; *HGS* hand grip strength; *hsCRP* high-sensitivity C-reactive protein; *LTM* lean tissue mass; *MAC* mid-arm circumference; *MAMA* mid-arm muscle area; *MAMC* mid-arm muscle circumference; *MIS* Malnutrition Inflammation Score; *NS* not significant; *SF-36* short-form (36-item) questionnaire; *SF-36 MCS* SF-36 mental health score; *SF-36 PCS* SF-36 physical health score; *TSF* triceps skinfold

^a^The number of patients in 'very poor' rating was very few (n = 2). Therefore data from 'very poor' and 'poor' ratings were merged

^b^Mean ± SD are provided for overall data. Appetite ratings data are reported as median with interquartile range (IQR)

^c^Kruskal-Wallis testing analysed significance across appetite ratings, Dunn post-hoc testing was carried for pair-wise comparisons between appetite ratings

^d, e, f, g, h^ same superscripts across appetite ratings indicate data were significantly different

All *P*-values <0.05 were indicative of significance

### PEW distribution as per appetite ratings

**Fig. 2:**
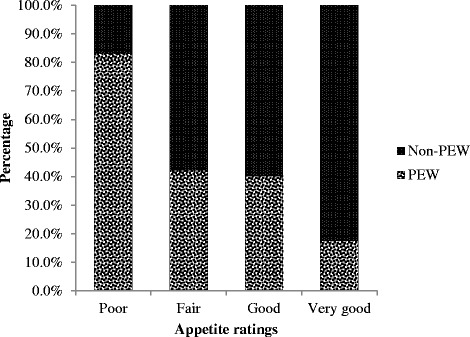
PEW distribution as per 4-scaled appetite ratings. Abbreviation: PEW = Protein energy wasting. Note-The number of patients in *very poor* rating was very few (*n =* 2). Hence, data in *very poor* and *poor* ratings were merged, thereby yielding the 4-scaled appetite ratings. The proportion of PEW patients increased significantly from 17.6 % in *very good* rating to 83.3 % in *poor* rating (*P* = 0.005, Pearson *χ* ^2^ test for trend, 2 cell counts less than 5)

Patients diagnosed with PEW were found in all appetite ratings with: 17.6 % (*n* = 6) in *very good*, 40.2 % (*n* = 35) in *good*, 42.3 % (*n* = 33) in *fair* and 83.3 % (*n* = 5) in *poor* ratings (Fig. 2). Based on Pearson *χ*^2^ analysis, the number of patients identified with PEW was significantly different across the appetite ratings (*P* = 0.005). However, the minimum expected count was less than 5 where the number of PEW patients in the *very good* and *poor* appetite ratings was very small. Hence, the 4-scaled appetite ratings were dichotomized to *normal* and *diminished* appetite categories.

Of the six patients who were rated as having *poor* appetite, five were diagnosed with PEW. The BMI for these five patients ranged between 19–22 kg/m^2^, serum albumin ranged between 30–46 g/dL and hsCRP ranged between 4.80-194.13 mg/L. In this group of six, one incidence of mortality was attributed to cardiac arrest six months post-data collection.

### Relationship between PEW diagnostic criteria as per dichotomized appetite categories

**Fig. 3:**
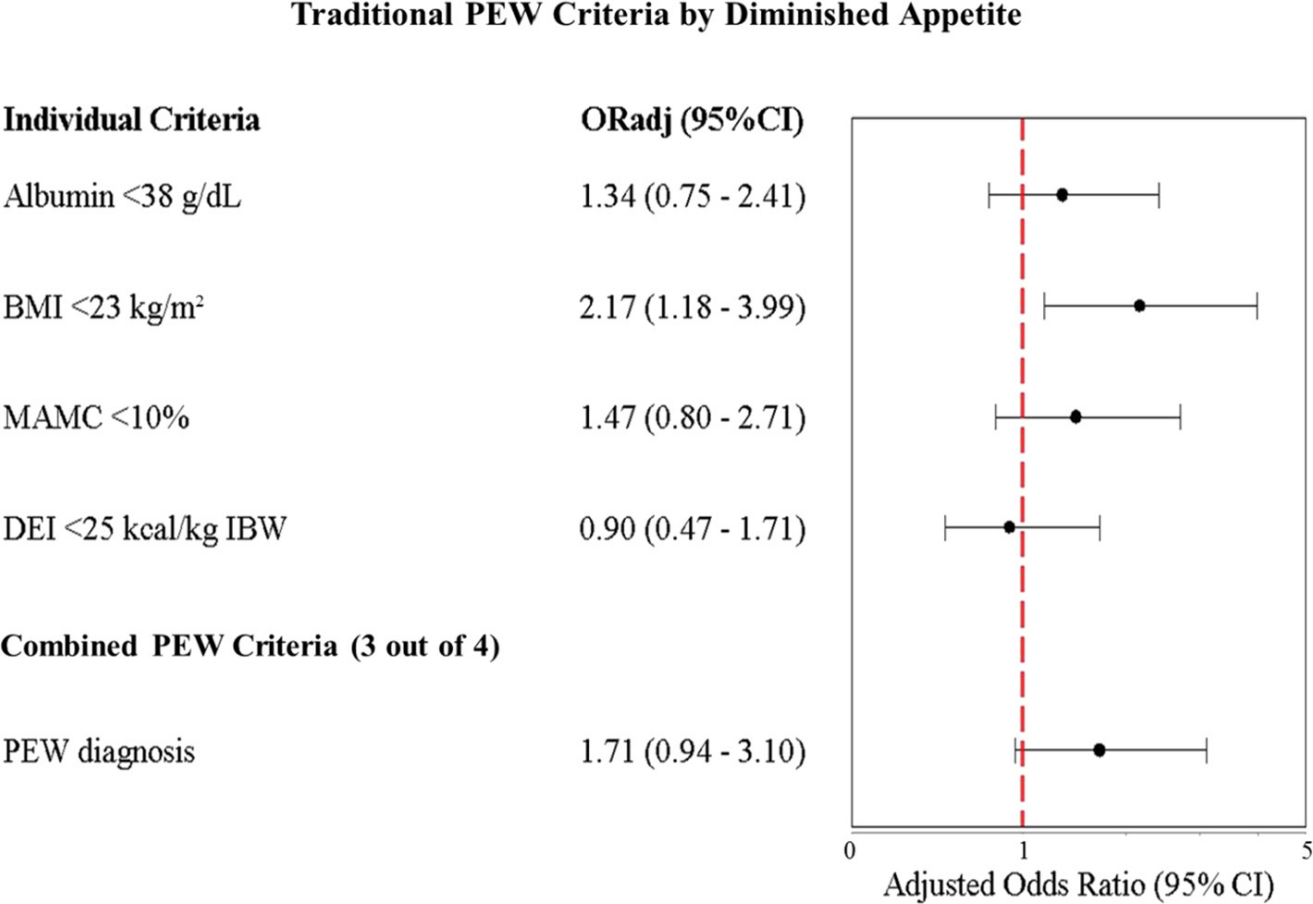
Adjusted odds ratio for patients with diminished appetite having PEW as per traditional PEW criteria. Abbreviations: OR_adj_ = adjusted odds ratio; BMI = Body Mass Index; CI = confidence interval; DEI = dietary energy intake; IBW = Ideal body weight; MAMC = mid-arm muscle circumference; PEW = protein energy wasting. ^*a*^ Patients were identified with PEW if fulfilling any 3 of 4 criteria for clinical diagnosis of PEW such as serum albumin <3.8 g per 100 ml (bromocresol green method), BMI <23 kg/m^2^, reduced MAMC (reduction >10 % in relation to 50th percentile of reference population) and unintentional low DEI <25 kcal/kg per/day for at least 2 months [7]. ^*b*^ The OR_adj_ were adjusted for age, gender, ethnicity, income level, co-morbidity and dialysis vintage by means of logistic regression analysis. ^*c*^ A vertical line represents odds ratio of 1. A value of 1 indicates no association between diminished appetite and PEW criteria, whether individual or combined. In the figure, the 95 % interval is also presented. If the value of 1 falls within the interval, there is no significant association between diminished appetite and PEW

**Fig. 4:**
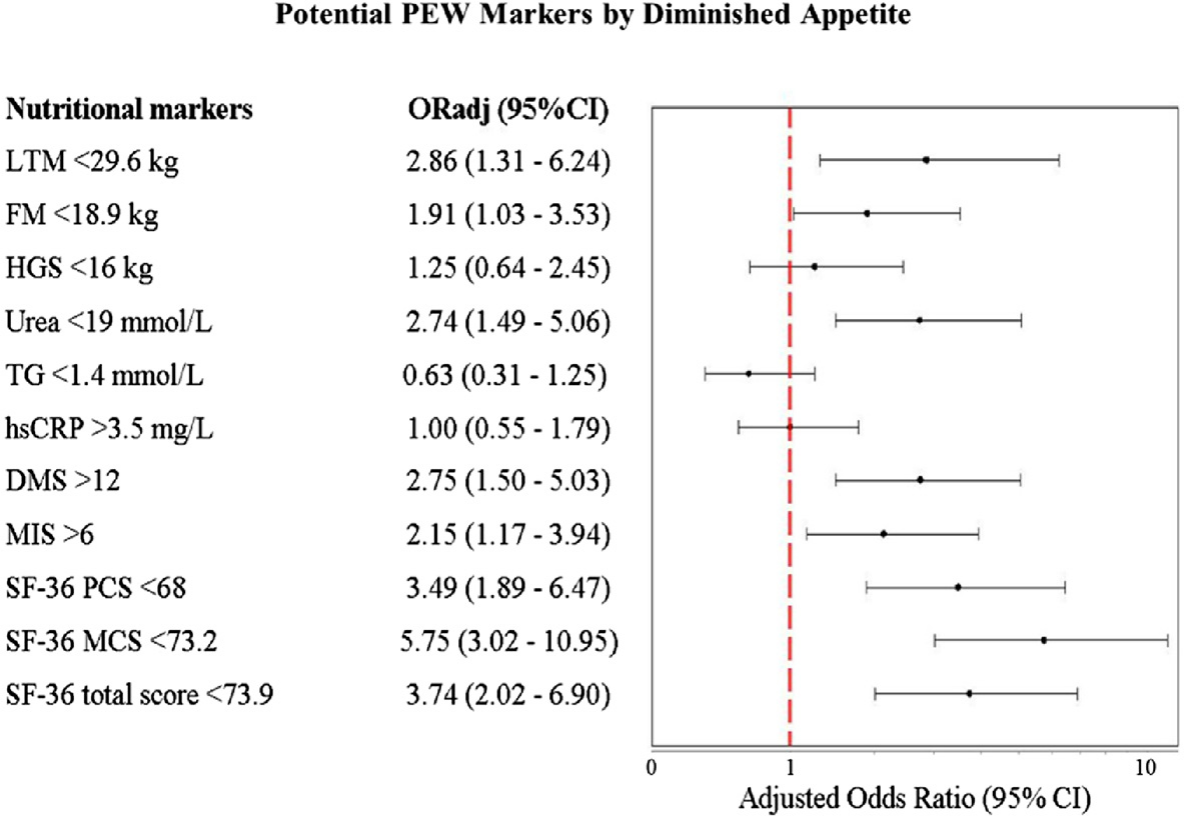
Adjusted odds ratio for patients with diminished appetite having PEW as per potential nutritional markers. Abbreviations: OR_adj_ = adjusted odds ratio; CI = confidence interval; DMS = Dialysis Malnutrition Score; FM = fat mass; HGS = hand grip strength; hsCRP = high sensitivity C-reactive protein; LTM = lean tissue mass; MIS = Malnutrition Inflammation Score; SF-36 = short-form (36-item) questionnaire; SF-36 MCS = SF-36 mental health score; SF-36 PCS = SF-36 physical health score; TG = serum triglycerides; TLC = serum total lymphocyte count. ^*a*^ Categorization for PEW assessment criteria were based on median of this population. ^*b*^ The OR_adj_ were adjusted for age, gender, ethnicity, income level, co-morbidity and dialysis vintage by means of logistic regression analysis. ^*c*^ A vertical line represents odds ratio of 1. A value of 1 indicates no association between diminished appetite and potential PEW markers. In the figure, the 95 % interval is also presented. If the value of 1 falls within the interval, there is no significant association between diminished appetite and potential PEW markers

Appetite ratings were dichotomized into 2 categories: normal appetite (*very good* and *good*) and diminished appetite (*fair* and *poor*). Associations between appetite categories with PEW prevalence based on traditional PEW diagnostic criteria as well as potential markers of PEW are presented in Figs. 3 and 4. The distribution of PEW patients in these two categories was similar [diminished, *n* = 38 (45.2 %); normal, *n* = 41 (33.9 %)] (see Additional file 1). Amongst the 4 traditional PEW diagnostic criteria, only BMI <23 kg/m^2^ showed significant association with appetite (*P* < 0.05) (Fig. 3). Patients reporting diminished appetite were more likely to have BMI <23 kg/m^2^ [adjusted odds ratio (OR_adj_: 2.17; 95 % CI: 1.18-3.99]. Diminished appetite had a marginal positive association with PEW diagnosis (OR_adj_: 1.71; 95 % CI: 0.94-3.10, *P* = 0.079). However, sensitivity (40–49 %), specificity (57–68 %) and accuracy (46–58 %) values for the various PEW diagnostic criteria compared to diminished appetite were below 80 % (see Additional file 1).

Alternately, potential markers of PEW such as serum urea and creatinine, LTM, DMS, MIS and SF-36 physical and mental component scores were significantly associated with dichotomized appetite categories (*P* < 0.05) (Fig. 4). Patients reporting diminished appetite were more likely to have urea <19 mmol/L (OR_adj_: 2.74; 95 % CI: 1.49–5.06) and creatinine <872 μmol/L (OR_adj_: 1.99; 95 % CI: 1.01–3.92). In terms of body composition, patients who reported diminished appetite were those with LTM <29.6 kg (OR_adj_: 2.86; 95 % CI: 1.31–6.24) and FM <18.9 kg (OR_adj_: 1.91; 95 % CI: 1.03–3.53). Patients reporting diminished appetite were more likely to score DMS >12 (OR_adj_: 2.75; 95 % CI: 1.50–5.03) and MIS >6 (OR_adj_: 2.15; 95 % CI: 1.17–3.94). As for QoL measures, patients with diminished appetite were more likely to score physical component score <68.0 (OR_adj_: 3.49; 95 % CI: 1.89–6.47) and mental component score <73.2 (OR_adj_: 5.75; 95 % CI: 3.02–10.95). Other potential markers of PEW such as hand grip strength, triglyceride, total lymphocyte count and hsCRP did not correlate with the diminished appetite category.

## Discussion

Studies have validated the first question from ADAT to assess appetite in dialysis patients, associating poorer appetite to poorer QoL, malnutrition, inflammatory markers, morbidity, increased hospitalization and increased mortality [6, 10, 12, 35]. When our patients were assessed using the 4-scaled appetite rating, only those with the poorer appetite ratings had significantly higher hsCRP and lower dry weight, BMI, MAC, MAMC, MAMA, LTM, serum urea and creatinine, dietary energy and protein intakes as well as SF-36 total scores. These findings were consistent with other studies [4, 10–12]. Patients reporting *very good* and *good* appetite had weight-adjusted energy and protein intakes below the recommendations of KDOQI Nutrition Guidelines that is 25 kcal/kg ideal body weight for energy intake and 1.2 g/kg ideal body weight for protein intake. These findings concur with the opinion that suboptimal nutritional intake is a phenomenon in the dialysis population [4, 37, 38]. Nutritional screening tools like DMS and MIS were found to correlate well with appetite assessment. However, we should note that this relationship is expected as appetite assessment is included as a component in these tools [12]. In contrast with other studies [4, 11, 38], serum albumin levels could not differentiate across appetite ratings for our HD patients. Altered serum albumin levels in chronic HD patients may be due to poor dietary intake, but most often metabolic acidosis and chronic inflammation may suppress albumin synthesis to a greater extent [39].

Diminished appetite is hypothesized to be related to PEW [6]. Logical thinking is that poor oral intake due to poor appetite would lead to malnutrition. Patients with prolonged poor appetite progressively develop malnutrition, invariably characterized by depleted muscle mass [40]. Malnutrition along with an increased resting energy expenditure, inflammation and metabolic acidosis, that are coexistent in chronic kidney disease, may contribute to the aetiology of PEW [5]. To the best of our knowledge, this study is the first to examine if subjective appetite reporting correlated to PEW prevalence in HD patients. We observed an increased trend in the percentage of PEW prevalence with poorer appetite ratings but owing to too few patient numbers reporting ‘poor’ appetite, a larger patient population is warranted to test this observation. After dichotomization, PEW prevalence was similarly distributed in both diminished and normal appetite categories. However, the adjusted odds ratio for the dichotomized appetite indicated that there was a marginal positive association between diminished appetite and PEW diagnosis. We found patients with diminished appetite were more likely to have lower LTM, serum urea and creatinine, nutritional status and QoL scores. This suggests that these markers were more indicative of poor oral intake. We believe that there is a possibility for genuine manifestations of anorexia-cachexia in our patients. Indeed, Bossola et al. [6] have reported anorexia to be present in one-third of dialysis patients. Kalantar-Zadeh et al. [12] reported anorexia to be present in 7 % of patients with “poor” appetite and 31 % in “fair” appetite categories.

Across the 4-scaled appetite ratings, our patients reported poor energy and protein intakes, as evident by the low EI: BMR ratio (<1.2). Resting energy expenditure has been noted to be different in PEW and non-PEW patients [41]. This observation suggests that PEW patients have elevated resting energy expenditure with decreased feeling of hunger whereas non-PEW patients with normal resting energy expenditure may experience a feeling of hunger [5]. Adaptive reduction in thermogenesis and feeling of hunger may have taken place in PEW patients in our study with prolonged diminished dietary intake, causing them to have a perception that their appetite was “normal”. Alternately, there were PEW patients who had acutely reduced food intake and were thus likely to report “diminished” appetite. Both these PEW groups could not experience hunger and thus fulfil model B as hypothesized by Carrero et al. [5]. There is likelihood that PEW patients who report normal appetite require intervention because they have developed mechanisms in adaptive thermogenesis and hunger feeling [5]. This may have masked the relationship of appetite as a reliable indicator of PEW in this patient population and would be regarded as a limitation to appetite assessment.

Studies report that diminished appetite is significantly associated with increased levels of inflammatory markers [4, 11, 12]. However, we did not find this association between dichotomized appetite categories and hsCRP levels. This could be explained by patients reporting ‘poor’ appetite also experiencing severe inflammation. This is evident by their hsCRP levels being 5 times higher compared to those who reported better appetite. It was observed that patients reporting better appetite ratings had uniformly similar levels of hsCRP. This perhaps explains why we could not establish appetite as a connective factor between PEW and inflammation, although inflammation has been suggested as contributory to PEW [5, 12].

Interestingly in the present study, we found that income was significantly associated with appetite ratings. The impact of culture on perceived health status should not be underestimated. Indeed, Angel and Thoits [42] have suggested that the *experience of illness is subjective and culture bound and therefore cognitive and linguistic categories of illness would be constrained in terms of interpretative and behavioral options in response to symptoms of illness*. Further, sick Malaysians are culturally ingrained to disclaim hunger or inability to eat or are even silent about the severity of illness. Behaviours contrary to these beliefs would be deemed impolite in a social structure without extensive medical insurance coverage or state health subsidies to cover medical health costs [3]. In these families, the burden of paying health bills, related to dialysis care, falls on the remaining earning household members. We found that patients reporting diminished appetite were 5.75 times more likely to have poorer mental scores for SF-36, which is likely caused by increased anxiety or depressive symptoms and emotional concerns [43, 44]. Ikizler et al. [45] recognize that a poor socioeconomic situation may be implicated in the matrix of inadequate nutrient intake and depression.

In line with literature, we found that appetite assessment correlated well with markers of nutritional status in this HD population. Given that appetite assessment has been proposed as a potential diagnostic tool for PEW by the ISRNM [7], we did observe a marginal positive association between diminished appetite and PEW. Therefore, this simple appetite question remains clinically relevant and may provide insights into poor oral intake in HD patients, serving as an early warning of impending malnutrition for nursing application where dieticians’ services are limited. Our study findings indicate that other potential diagnostic markers of PEW may lend greater evidence to linking appetite to PEW. Further research to elucidate PEW diagnostic criteria related to appetite assessment is warranted.

As this was a cross-sectional study, appetite assessment was only carried out once and we cannot elucidate the cause-and-effect between appetite and development and/or progression of PEW. We recommend that in order to have a more reliable understanding on appetite, the appetite assessment should be repeated over time. Furthermore, as this study only included patients from urban dialysis centres, we may not be able to extrapolate these observations to patients from rural dialysis centres.

## Conclusions

Appetite assessment was consistent in linking diminished appetite with declining measures of nutritional status in the Malaysian haemodialysis population. A graded but non-significant increase in the proportion of PEW patients occurred as appetite became poorer. However, after dichotomization, a marginal positive association was observed between diminished appetite and PEW. Future mechanistic and longitudinal studies are needed to confirm this association and assess whether early detection and correction of diminished appetite could improve the nutritional status and subsequently reduce the PEW occurrence in HD patients.

## Additional file

Additional file 1:
**Sensitivity, specificity and accuracy of PEW diagnostic criteria according to dichotomized appetite categories.**

## Abbreviations

ADAT: Appetite and Dietary Assessment Tool
BMI: Body mass index
CI: Confidence interval
DMS: Dialysis Malnutrition Score
EI:BMR: Energy intake to basal metabolic rate
FM: Fat mass, HD, haemodialysis
hsCRP: High-sensitivity C-reactive protein
ISRNM: International Society of Renal Nutrition and Metabolism
KDOQI: Kidney Disease Outcomes Quality Initiative
Kt/V: Dialysis dose
LTM: Lean tissue mass
MAC: Mid-arm circumference
MAMA: Mid-arm muscle area
MAMC: Mid-arm muscle circumference
MIS: Malnutrition Inflammation Score
OR_adj_: Adjusted odds ratio
PEW: Protein energy wasting
QoL: Quality of life
SF-36: 36-item short form health survey
TSF: Triceps skinfold
