# Supplementary material for: Assessing protein energy wasting in a Malaysian haemodialysis population using self-reported appetite rating: a cross-sectional study
Source: BMC Nephrol. 2015 Jul 7;16:99. doi: 10.1186/s12882-015-0073-x (PMC4492004; doi:10.1186/s12882-015-0073-x)
Supplement: Additional file 1: — Sensitivity, specificity and accuracy of PEW diagnostic criteria according to dichotomized appetite categories. [file 12882_2015_73_MOESM1_ESM.pdf]

Additional file 1: Sensitivity, specificity and accuracy of PEW diagnostic criteria according to appetite categories

**PEW criteria**[a]	**Diminished appetite (n=84)**[b]	**Normal appetite (n=121)**[b]	**Sensitivity (%)**	**Specificity (%)**	**Accuracy (%)**
Serum albumin< 38 g/dL	45 (53.6)	54 (44.6)	45.5	63.2	54.6
BMI <23 kg/m^2	52 (61.9)	54 (44.6)	49.1	67.7	58.0
MAMC < 10%	51 (60.7)	64 (52.9)	44.3	63.3	52.7
DEI < 25kcal/kg IBW	53 (63.1)	80 (66.1)	39.8	56.9	45.9
PEW diagnosis	38 (45.2)	41 (33.9)	48.1	63.5	57.6

Footnotes:

Abbreviations: BMI = Body mass index; DEI = dietary energy intake; IBW = ideal body weight, MAMC = mid-arm muscle circumference; PEW = protein energy wasting

[a] PEW criteria and its cut-offs was provided by the International Society of Renal Nutrition and Metabolism (ISRNM) panel such as serum albumin <3.8 g per 100ml (bromocresol green method), BMI <23 kg/m^2, reduced MAMC (reduction >10% in relation to 50th percentile of reference population) and unintentional low DEI <25kcal/kg per/day for at least 2 months [7].

[b]Data for appetite categories are presented as frequency (%)
